# A Rare Case of an Intraductal Papillary Mucinous Neoplasm of Pancreas Fistulizing Into Duodenum With Adult Polycystic Kidney Disease

**DOI:** 10.14740/gr657w

**Published:** 2015-04-03

**Authors:** Nirav Pipaliya, Chetan Rathi, Pathik Parikh, Ruchir Patel, Meghraj Ingle, Prabha Sawant

**Affiliations:** a Department of Gastroenterology, Lokmanya Tilak Municipal Medical College and General Hospital, Sion, Mumbai, India

**Keywords:** Fistulizing IPMN, ADPKD, Duodenum

## Abstract

Intraductal papillary mucinous neoplasm (IPMN) accounts for 20-50% of all cystic neoplasms of the pancreas. Rarely, IPMN, whether benign or malignant, can fistulize into adjacent organs like duodenum, stomach or common bile duct. IPMN can be associated with other diseases like Peutz-Jeghers syndrome and familial adenomatous polyposis. Association with adult polycystic kidney disease (ADPKD) is extremely rare. We report a case of a 60-year-old male with a large IPMN in the head of the pancreas diagnosed by magnetic resonance imaging, endoscopic ultrasound and cyst fluid analysis. It was complicated by fistula formation into the second part of the duodenum. Patient was simultaneously having adult polycystic kidney disease. There is only one case report of uncomplicated IPMN with ADPKD in the literature so far. And even rarer, there is no any case report of fistulizing IPMN with ADPKD reported so far, to the best of our knowledge.

## Introduction

Cystic neoplasms of the pancreas are rarely encountered, diagnosed in 10% of pancreatic cysts detected on imaging. They consist of intraductal papillary mucinous neoplasm (IPMN) of the pancreas, mucinous cystic neoplasm, serous cystadenoma, papillary cystic tumors and cystic islet cell tumors [1]. IPMN accounts for 1-3% of all exocrine pancreatic neoplasms and 20-50% of all cystic neoplasms of the pancreas [2].

Though rare, IPMN, whether benign or malignant, can fistulize into adjacent organs like duodenum or stomach. Moreover, IPMN can be associated with many other conditions like Peutz-Jeghers syndrome and familial adenomatous polyposis but association with adult polycystic kidney disease (ADPKD) is extremely rare and is almost unheard of. Only one case of IPMN (non-complicated) with ADPKD has been reported in the literature [3].

We report a case of a 60-year-old male diagnosed to have an IPMN complicated by a fistula formation into second part of the duodenum associated with ADPKD. There is not a single case report of similar combined presentation in an individual patient reported in the literature so far, to the best of our knowledge.

## Case Report

A 60-year-old male, chronic smoker, non-alcoholic, presented with 3 months history of pain in upper abdomen which was epigastric, dull aching, continuous, mild to moderate intensity and radiating to back. He had significant anorexia and weight loss of 8 kg. He did not have similar pain in the past. On physical examination, the patient was pale, malnourished, afebrile, with a blood pressure of 150/90 mm Hg, pulse rate of 90 beats per minute and a respiratory rate of 22 breaths per minute. Abdominal examination revealed soft and non-tender abdomen with non-palpable liver and spleen. There was an approximately 8 × 8 cm ballotable non-tender cystic lump with ill defined margins noted in left lumber region which did not move with respiration.

Laboratory examination showed hemoglobin of 9.2 g/dL, total leukocyte count 8,800/mm^3^, and platelet count of 3.3 lakh/mm^3^. Renal function tests showed serum creatinine of 3.3 mg/dL and blood urea nitrogen of 54 mg/dL. Liver function tests were normal. Serum amylase and lipase were 90 IU/L and 80 IU/L, respectively. Serum CA 19-9 was 20 IU/mL.

**Figure 1:**
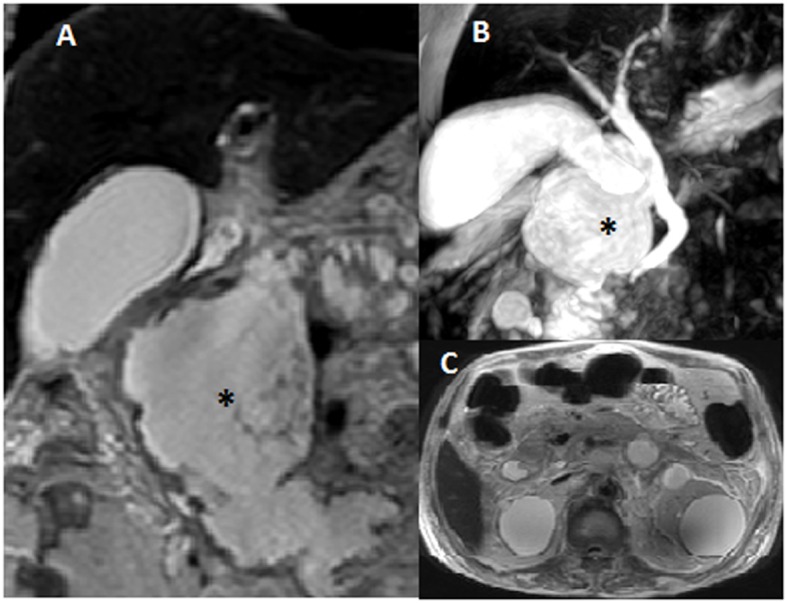
Magnetic resonance cholangiopancreatography with MRI abdomen with pelvis showed large 8 × 5 × 5 cm cystic irregular collection (asterisk) with solid component and septations involving head and body of the pancreas, almost replacing them (A, B). Bilateral multiple cysts in kidneys were also noted (C).

**Figure 2:**
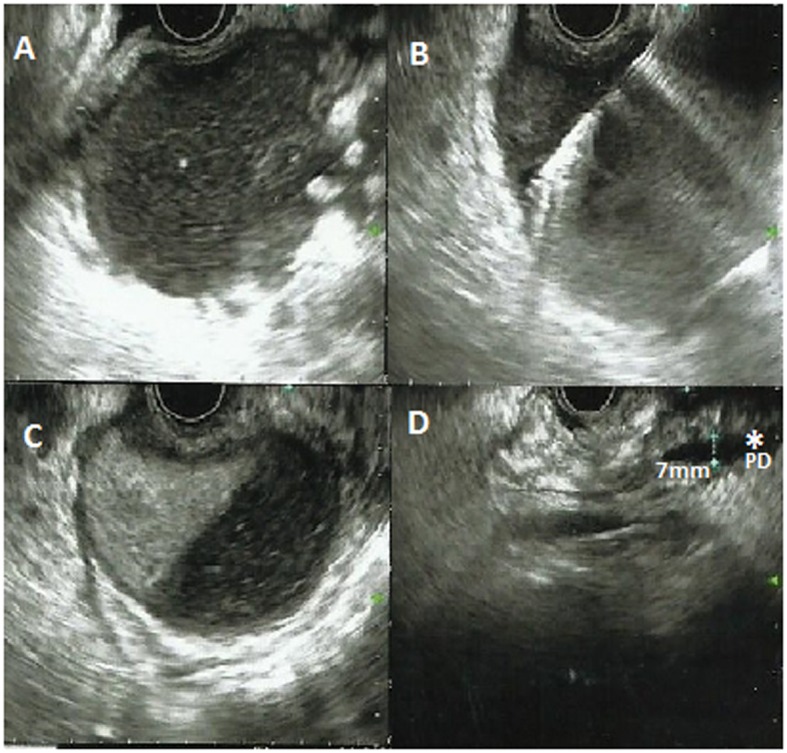
Endoscopic ultrasound revealed ill defined cystic lesion in the head of the pancreas with some solid component (A, B, C). EUS guided needle aspiration was taken (B). Pancreatic duct was dilated (7 mm) (asterisk) (D). The lesion was communicating with the second part of duodenum.

**Figure 3:**
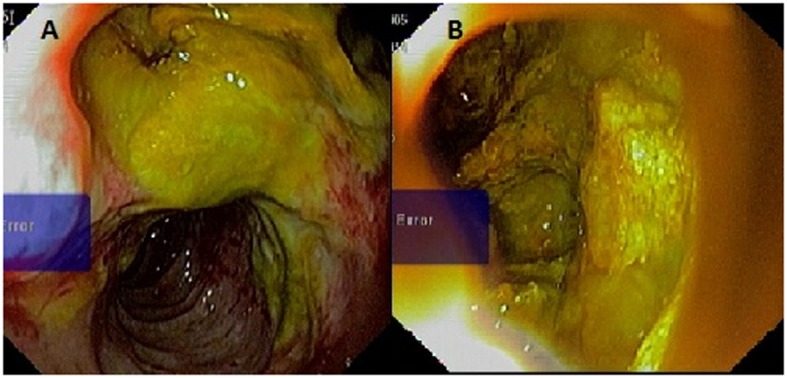
Esophagoduodenoscopy showing an fistulous opening in the second part of duodenum just distal to the papilla. Opening was discharging mucus and debris (A). Tumor cavity with solid component was seen from the fistulous opening (B).

Ultrasonography of the abdomen was suggestive of dilated pancreatic duct (7 mm in head) and a 6 × 5 × 5 cm well defined cystic collection with wall thickness of 4 mm with multiple mobile internal echoes seen in the head and body of pancreas which was communicating with pancreatic duct. It also showed bilateral innumerable renal cortical cysts, largest measuring 10 × 10 cm in left kidney. Magnetic resonance cholangiopancreatography (MRCP) with magnetic resonance imaging (MRI) abdomen showed large 8 × 5 × 5 cm cystic irregular collection with septations involving head and body of the pancreas, almost replacing them with 7.5 mm pancreatic duct. MRI also suggested bilateral polycystic kidney disease (Fig. 1). Endoscopic ultrasound (EUS) suggested ill defined cystic lesion in the head of the pancreas with some solid component and 7 mm pancreatic duct (Fig. 2). Second part of duodenum seemed to be ulcerated and the cyst was in communication with D2. EUS guided aspiration of pancreatic cyst fluid revealed high viscosity fluid with fluid amylase 945 IU/L and fluid CEA of 312 ng/mL. Cytology revealed clusters of columnar epithelial cells with mild degree of dysplasia in a background of mucin-like material, histiocytes and cellular debris. Subsequent esophagogastroduodenoscopy revealed a fistulous opening on the medial wall of the second part of duodenum (D2) just distal to the papilla with infiltrated mucosa. The opening was discharging mucus material and necrotic debris. The tumor cavity was visible through the opening (Fig. 3). Biopsy from the fistulous tract was taken. It turned out to be negative for malignancy. We referred the patient for pancreaticoduodenectomy. But the patient was declared high risk by the anesthetist in view of chronic renal failure, hypertension, poor respiratory reserve and ischemic heart disease. Patient chose not to undergo surgery.

## Discussion

IPMN is defined as an intraductal epithelial tumor composed of mucin-producing columnar cells showing papillary proliferation, cyst formation, and variable degrees of cellular atypia, even within an individual neoplasm.

IPMN, whether benign or malignant, can become complicated in 6.6% cases forming a fistula into adjacent organs [4]. The fistulizing behavior is of two types: penetrating/invasive type and automatic/mechanical type. In the penetrating/invasive type, tumor cells invade the adjacent organs by forming a malignant fistula. This pattern is seen mostly in malignant tumors only. In the automatic type, rising intratumoral pressure causes the tumor to rupture into adjacent organ releasing the content into it [5]. This type is seen mostly in benign IPMN, though a large malignant IPMN can also have this type of fistula. Kobayashi et al reported that automatic/mechanical type was accounted for 67% cases of fistulizing IPMN [4]. In our case, biopsy from fistula site in the duodenum was negative for malignancy. So, it was classified as automatic type rupture resulting in fistula formation.

The most common organ fistulized is the duodenum (64%), followed by the common bile duct (56%) and the stomach (17%) [5]. Few case reports of intraperitoneal rupture resulting in pseudomyxoma peritonei have been reported [6]. The 5-year survival rates of patients with benign and malignant IPMNs have been reported to be 85-100% and 25-65%, respectively. However, the 5-year survival of patients with IPMNs fistulizing into other organs cannot be calculated because the case reports of fistulizing IPMN are rare according to the literature review.

Association of IPMN with ADPKD is very rare. Only one case has been reported in 2009 by Yasunori Sato from Japan. Simple pancreatic cysts can be found in as many as 7-10% patients with ADPKD, but rarely a cyst in the pancreas can turn out to be a cystic neoplasm of the pancreas. Other diseases that are associated with IPMN are Peutz-Jeghers syndrome and familial adenomatous polyposis [7].

In case of fistulizing IPMN, a biopsy specimen should be taken from the fistula endoscopically which would reveal whether the tumor has invaded an organ adjacent to the pancreas and enable determination of extent of surgery and prognosis. There are no clear guidelines to manage IPMN complicated by a fistula formation. However, pancreaticoduodenectomy is the standard of care for an IPMN fistulizing into duodenum in most of the centers.

Our patient had a large IPMN involving the head of the pancreas diagnosed on MRI, EUS and cyst fluid analysis with unusual feature of fistula formation into second part of duodenum and simultaneous association with ADPKD. This is the first case, to the best of our knowledge, of a large IPMN with ADPKD and a fistula formation into duodenum.
